# Treatment of post-traumatic osteochondral lesions of the talus: a four-step approach

**DOI:** 10.1007/s00167-012-2028-0

**Published:** 2012-05-10

**Authors:** Alberto Ventura, Clara Terzaghi, Claudio Legnani, Enrico Borgo

**Affiliations:** 1 U.O.S.D. Chirurgia Articolare Mininvasiva (Minimally Invasive Articular Surgery Unit), Istituto Ortopedico G. Pini, Milan, Italy; 2 Scuola di Specializzazione in Ortopedia e Traumatologia, Università degli Studi di Milano, Milan, Italy

## Abstract

**Purpose:**

The purpose of this retrospective study was to assess the treatment of post-traumatic osteochondral lesions (OCLs) of the ankle with a four-step protocol.

**Methods:**

Thirty-eight patients with at least one MRI-documented OCL of the ankle were treated from 2004 to 2010. Median age at surgery was 39 years (range: 18–52). Mean lesion size was 1.0 cm^2^ (SD: 0.2). All patients underwent a four-step surgical procedure including synovectomy, debridement and microfractures of the OCL, capsular shrinkage, and bracing and non-weightbearing for 21 days. Clinical assessment included objective examination, the AOFAS ankle and hindfoot scoring system, Karlsson-Peterson score, Tegner activity level, and Sefton articular stability scale. MRI scans were taken 18 months after surgery in all patients.

**Results:**

Follow-up examination at an average of 4 years (SD: 1.1) after surgery showed significant improvement of all variables compared to pre-operative values (*P* < 0.05). Most patients rated their outcome as good/excellent. MRI scans taken 18 months after surgery documented completely repaired lesion in 27 ankles, slight bone marrow oedema with partially repaired defect in 9 patients, and visible defect in 2 ankles.

**Conclusion:**

Based on the present results, we propose a comprehensive four-step protocol as a safe and clinically effective treatment option in patients with post-traumatic OCLs of the ankle.

**Level of evidence:**

Retrospective case series, Level IV.

## Introduction

Several therapeutic strategies exist for the treatment of osteochondral lesions (OCLs) of the talus [22, 37]. Non-operative treatment consists of cast immobilization and non-weightbearing or weightbearing as tolerated along with a rehabilitation programme [13, 26]. Recently, platelet-rich plasma and hyaluronic acid injections have also been employed for treating talar cartilage defects [21].

Common surgical techniques include arthroscopic debridement with curettage and drilling [3, 19], arthroscopic debridement with microfractures [6, 8], autologous chondrocyte transplantation [4, 5], autologous matrix-induced chondrogenesis [7, 33], and arthrotomy with osteochondral autologous or heterologous grafts [1].

The advantages of arthroscopic procedures for the treatment of OCLs in the ankle are that they minimize invasiveness, reduce operating time, and allow a faster rehabilitation period and an earlier return to work in comparison with open procedures [10, 11, 23].

In the young and active population, OCLs following sports-related injuries are usually associated with the involvement of the external ligamental complex and with concomitant synovitis [24, 28, 30, 35], and thus, all aspects should be addressed during the treatment in order to maximize the outcomes.

A four-step protocol, which consists of synovectomy, debridement and microfractures of the OCL, capsular shrinkage, and bracing and non-weightbearing, has been developed in the Department of Minimally Invasive Articular Surgery of the G. Pini Orthopaedic Institute of Milan, Italy. The purpose of the present study was to retrospectively evaluate the outcomes of patients with sports-related OCLs of the talus. The hypothesis was that a comprehensive four-step approach would lead to a high success rate in patients affected by post-traumatic OCLs of the ankle.

## Materials and methods

Forty patients with OCLs of the talus were treated from 2004 to 2010. Operative treatment was proposed to subjects with deep ankle pain symptoms during daily living or sports activities who were unresponsive to at least 6 months of non-operative treatment including physical therapy and proprioceptive training. Diagnosis was confirmed by magnetic resonance imaging (MRI). All the operations were performed by the same experienced senior surgeon. All subjects considered met the following inclusion criteria: age 18–55 years, absence of previous ankle surgery, absence of multiple ligament insufficiency, cartilage defects less than 1.5 cm^2^.

### Surgical technique

Operative setting and preliminary arthroscopic inspection were made according to those described by the authors in a previous paper [36].

**Fig. 1:**
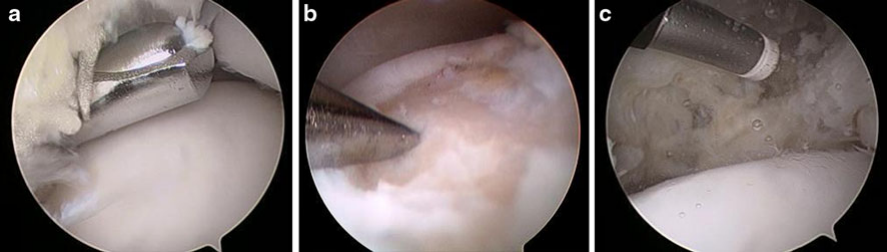
Operative procedure, including synovectomy (**a**), debridement and microfracture of the OCL (**b**), and capsular shrinkage (**c**)

Synovectomy was performed whenever hypertrophic synovitis was detected (Fig. 1a). Cartilage defects were first debrided with the use of a motorized 4.0-mm shaver (Tomcat, Stryker Endoscopy, San Jose, CA, USA; Formula, Stryker Endoscopy, San Jose, CA, USA).

After unstable cartilage had been excised and the overlying bone had been scraped, the defect area was curetted, and the area of the defect was perforated according to the technique described by Steadman [27] (Fig. 1b).

The shrinkage procedure was performed using a VAPR T Side Effect Thermal Electrode (Mitek, Westwood, MA, USA) with a 3.5-mm tip at desication mode, a temperature setting of 70 °C, and maximum power of 50 W. With the ankle placed in an everted position, the probe was swept from the anterior talo-fibular ligament (ATFL) and progressing along the adjacent capsule based on visual observation of the macroscopic tissue contraction (Fig. 1c). Whenever the ATFL presented a complete tear, ligament remnants were debrided before performing thermal shrinkage on the surrounding capsule in the same fashion as described by the authors in their previous study [36].

Associate procedures such as the removal of loose bodies or osteophytes were performed if required. At the end of the procedure, the tourniquet was released to visualize bleeding of the OCL site, joint flushing was performed, portals were closed, and a sterile dressing was applied.

Immediately after the operation, an ankle brace was applied (Air-Stirrup, Aircast Inc., Summit, NJ, USA). For the first 3 weeks, patients were instructed to walk with non-weightbearing with the use of two crutches. After the removal of the brace, patients were encouraged to regain weightbearing as tolerated and were instructed to perform proprioceptive and complete ankle range of motion (ROM) exercises. Return to sports was permitted 12 weeks post-operatively.

### Outcome measures

Patients were examined pre-operatively and followed up after an average time of 4 years (SD: 1.1). Clinical assessment included the American Orthopaedic Foot and Ankle Society (AOFAS) ankle and hindfoot scoring system [18], Karlsson-Peterson score [17], Tegner activity level [31], Sefton articular stability scale [25], and objective examination comprehending ROM, anterior drawer sign [34], and talar tilt test [13]. Both anterior drawer and talar tilt tests were performed manually by the same investigator. The test was considered either positive (maximum manual translation more than 5 mm) or negative (manual translation less than 5 mm). Magnetic resonance imaging (MRI) scans were taken in all patients 18 months after surgery. Patients were also asked to rate the success of their surgery as poor, fair, good, or excellent and to indicate whether they would undergo surgery again.

### Statistical analysis

Data were analysed using the program SPSS Version 17.0 (SPSS Inc., Chicago, IL, USA). Paired t-test (two-sided test and α = 0.05) was utilized to compare the pre-operative and follow-up status. Differences with a *P* value <0.05 were considered statistically significant.

## Results

One patient suffered further malleolar fracture on the operated ankle that was treated surgically, and one patient was lost at follow-up; thus, 38 ankles were considered at follow-up. Patient demographics are reported in Table 1. Mean size of the lesion was 1.0 cm^2^ (SD: 0.2). The defects were mostly located in the anteromedial portion of the talus (31 ankles, 82 %), while in 7 cases (18 %) they were located in the posterolateral talar region. Fourteen patients practised contact sports before injury (martial arts, rugby, soccer, basketball, etc.), and 24 non-contact sports (volleyball, running, tennis, etc.). Twelve practised sport at a competitive level, five of them as professionals, and the other 26 practised sport at the recreational level.

**Table 1:** Patient demographics and anthropometric data

Median age at surgery		39 years (range: 18–52)
Gender	Male	23
	Female	15
Side	Left	17
	Right	21
Mean Body Mass Index (BMI)		26 (SD: 3)

An overview of the results is presented in Table 2. The mean AOFAS and Karlsson-Peterson score significantly improved at follow-up (*P* < 0.001), as well as Tegner activity level (*P* < 0.001). Return to sports occurred at an average of 15.4 weeks (SD 3.1) after surgery. Articular stability as assessed by Sefton scale documented a significant improvement (*P* < 0.001).

**Table 2:** Overview of the results of clinical assessment

	Pre-operative	Post-operative	*P* value
AOFAS Scale (mean)	60.2 (SD: 7.8)	89. 4 (SD: 7.1)	(< 0.001)
Karlsson-Peterson score (mean)	62.7 (SD: 9.9)	90.1 (SD: 7.8)	(< 0.001)
Tegner score (median)	4 (range 2–5)	4 (range 3–5)	(< 0.001)
Sefton scale (mean)	2.9 (SD: 0.9)	1.8 (SD: 0.8)	(< 0.001)
Positive anterior drawer test	30 (79 %)	1 (2.6 %)	(< 0.001)

Twenty patients (52 %) complained giving-way symptoms before surgery. Anterior drawer test was positive in 30 (79 %) patients. None of the patients was positive to talar tilt test. Objective examination documented a statistically significant improvement in terms of ankle stability and demonstrated negative anterior drawer test in 37 (97.4 %) patients. Results were compared to pre-operative manual laxity tests (*P* < 0.001). ROM was complete in 36 patients: one experienced 5 degrees dorsiflexion limitation and one patient 10 degrees less compared to the contralateral side. None reported swelling.

Concomitant arthroscopic findings are summarized in Table 3: most notably, ATFL was found partially or totally ruptured in all subjects.

**Table 3:** Overview of concomitant arthroscopic findings and related treatment

Pathology	No. of patients	%	Treatment
Synovitis	38	100	Synovectomy
ATFL lesion	38	100	Debridement and shrinkage
Loose bodies	7	18	Removal
Tibiotalar bony impingement	5	13	Bony spurs resection

*ATFL* anterior talo-fibular ligament

Complications that persisted at follow-up were observed in two patients (5.3 %) who complained of permanent altered sensation of the anterolateral aspect of the foot.

MRI scans taken at 18 months after surgery revealed filling of the defect in 27 (71 %) cases.

In 9 ankles (24 %), the treated area appeared partially repaired with slight bone marrow oedema which was still present. In 2 ankles (5 %), visible defect was still present together with subchondral abnormalities.

Thirty-one patients (82 %) rated the success of their surgery as good/excellent. Eight patients (18 %) judged the result as fair. All patients stated they would have undergone surgery again.

## Discussion

The most important finding of the present study was that the four-step technique presented is a valid therapeutic option for the treatment of osteochondral defects, since it contributes to fibrocartilage regeneration and confirms subjective and objective clinical improvement over a period of up to 6 years after surgery. Secondly, the clinical and functional results of this study are similar or superior to those previously reported in patients treated for OCLs of the talus with or without associated ankle instability, with no increased risk of complications.

Recently, several studies documented successful outcomes after bone marrow stimulation for the treatment of ankle OCLs [6, 8, 14, 27, 32], with optimal results observed in lesions smaller than 15 mm and in patients without concomitant factors that could negatively affect outcomes (increasing age, higher body mass index, history of trauma, and presence of osteophytes) [8].

The four-step operative treatment included synovectomy (Figure 1a), debridement and microfracturing of the OCL (Fig. 1b), ATFL and capsular shrinkage (Fig. 1c), and bracing and non-weightbearing for 21 days.

In the sprained ankle, synovitis is a common finding as it represents the reaction to an insult to the lateral ligament complex [30]. Synovial hypertrophy was detected and treated in all patients who had suffered previous ankle sprain, similar to how described in a previous study by the same authors [36].

A strong association between OCLs and ankle instability in athletes has been previously reported [24]. In all the ankles examined in this case series, the ATFL was found either thickened or avulsed during arthroscopic inspection. The resection of the borders of the avulsed ligament and the debridement of the adjacent area allow the stimulation of fibrous tissue and enhancement of the healing process of the ATFL complex. This procedure, together with the thermal shrinkage provided by radiofrequency, promotes the shortening of collagenous fibres within the connective tissue and leads to an effective reduction in capsular volume, thus enhancing joint stability [2, 9, 12].

Rehabilitation protocol consisted of bracing with non-weightbearing for 3 weeks.

Controversy exists regarding the concomitant treatment of OCLs and lateral ankle instability, since different rehabilitation programmes exist for the two treatment strategies. Patients treated with microfractures would need early ankle motion to promote fibrocartilage healing, while immobilization with cast or brace is crucial to prevent lengthening of the treated tissue after thermal shrinkage [15]. Other papers addressing combined lateral ligament reconstruction and treatment of OCLs report cast immobilization for three [15] or 4 weeks [29]. In this case series, the positioning of the Air-Stirrup brace during the first 3 weeks of the post-operative phase permitted the movement on the sagittal plane only, thus allowing earlier mobilization of the ankle without affecting ligament healing.

In the patient group considered, the positive results observed in subjective perception and functional scales were also confirmed by objective examination. At follow-up, all patients reported a reduction in pain and a functional improvement. These results are in agreement with previously reported results [15, 29].

Lee et al. [20] reported on 35 ankles with isolated OCLs of the talar dome which were treated with arthroscopic microfractures. At a mean follow-up of 33 months, mean AOFAS score was 90 points. However, differently from the cohort of patients reported in the present paper, no instability was found in any of the ankles considered. Takao et al. [29] performed arthroscopic drilling of OCLs of the talus in 69 unstable ankles that were treated with lateral ankle ligament reconstruction. Overall mean AOFAS score reached 94.5 points at 1 year after surgery. Gregush and Ferkel [15] reported on 31 patients who underwent concomitant arthroscopic treatment of an OCL and lateral ankle stabilization in the same sitting after an average follow-up of 7.3 years. The mean AOFAS score was 89 with no patients complaining of ankle instability at the time of follow-up.

In the present study, MRI scans taken 18 months after surgery documented complete filling of the defect in 71 % of cases. The MRI scans for two patients (5 %) reported incomplete filling of the defect.

After 4 years, none of the patients expressed symptoms of giving way or any other complaint of secondary instability. In none of the subjects was there noted persistent deep ankle pain affecting daily activities and causing joint swelling after sport or strenuous activity. Three patients (8 %) reached a lower activity level at follow-up. These patients did not return to their previous sport activity because of fear of reinjury.

No cases of wound infections nor recurrent instability was reported. In a previous paper on arthroscopic treatment of ankle instability by the same authors, 5 cases (5.7 %) of peroneal nerve injury persisting at follow-up were reported. In the present study, occurrence of neurologic complications was found to be a little lower (5.3 %). However, these complications did not considerably affect the overall functional outcome.

Limitations of the present study include its retrospective nature, the lack of a control group, and the relatively small sample size. The limited number of patients is due to the fact that this approach requires adopting highly selective indications as criteria for patient selection.

Osteochondral talar dome lesions are strictly related to soft tissue pathology, and they may coexist because of the mechanism of injury [16, 28]. Arthroscopic microfracturing itself could be insufficient for the treatment of sports-related OCLs of the ankle since usually the presence of cartilage damage implies concomitant involvement of the lateral ligament complex. The four-step procedure as suggested represents a comprehensive approach that can lead to reliable improvement in symptoms in the treatment of post-traumatic OCLs ankle lesion in appropriately selected patients. In addition, the four-step approach has multiple advantages compared to open techniques (low complication rate, less operative time, no donor site morbidity) and offers similar outcomes in comparison with previously reported case series. Further randomized trials are needed to substantiate these findings.

## Conclusion

A comprehensive four-step protocol as presented represents a safe and clinically effective treatment option in patients with post-traumatic OCLs of the ankle.

## References

[1] Al-Shaikh RA, Chou LB, Mann JA, Dreeben SM, Prieskorn D (2002). Autologous osteochondral grafting for talar cartilage defects. Foot Ankle Int.

[2] Arnoczky SP, Aksan A (2002). Thermal modification of connective tissues: basic science considerations and clinical implications. J Am Acad Orthop Surg.

[3] Barnes CJ, Ferkel RD (2003). Arthroscopic debridement and drilling of osteochondral lesions of the talus. Foot Ankle Clin.

[4] Battaglia M, Vannin F, Buda R, Cavallo M, Ruffilli A, Monti C, Galletti S, Giannini S (2011). Arthroscopic autologous chondrocyte implantation in osteochondral lesions of the talus: mid-term T2-mapping MRI evaluation. Knee Surg Sports Traumatol Arthrosc.

[5] Baums MH, Heidrich G, Schultz W, Steckel H, Kahl E, Klinger HM (2006). Autologous chondrocyte transplantation for treating cartilage defects of the talus. J Bone Jt Surg Am.

[6] Becher C, Driessen A, Hess T, Longo UG, Maffulli N, Thermann H (2010). Microfracture for chondral defects of the talus:maintenance of early results at midterm follow-up. Knee Surg Sports Traumatol Arthrosc.

[7] Benthien JP, Behrens P (2011). The treatment of chondral and osteochondral defects of the knee with autologous matrix-induced chondrogenesis (AMIC): method description and recent developments. Knee Surg Sports Traumatol Arthrosc.

[8] Chuckpaiwong B, Berkson EM, Theodore GH (2008). Microfracture for osteochondral lesions of the ankle: outcome analysis and outcome predictors of 105 cases. Arthroscopy.

[9] Cline S, Wolin P (2001). The use of thermal energy in ankle instability. Clin Sports Med.

[10] Easley ME, Latt LD, Santangelo JR, Merian-Genast M, Nunley JA (2010). Osteochondral lesions of the talus. J Amer Acad Orthop Surg.

[11] Ferkel RD, Zanotti RM, Komenda GA, Sgaglione NA, Cheng MS, Applegate GR, Dopirak MR (2008). Arthroscopic treatment of chronic osteochondral lesions of the talus: long-term results. Am J Sports Med.

[12] Flory PJ, Garrett RR (2002). Phase transition in collagen and gelatine systems. Am J Chem Soc.

[13] Gaebler C, Kukla C, Breitenseher MJ, Nellas ZJ, Mittlboeck M, Trattnig S, Vécsei V (1997). Diagnosis of lateral ankle ligament injuries. Comparison between talar tilt, MRI and operative findings in 112 athletes. Acta Orthop Scand.

[14] Gobbi A, Francisco RA, Lubowitz JH, Allegra F, Canata G (2006). Osteochondral lesions of the talus: randomized controlled trial comparing chondroplasty, microfracture, and osteochondral autograft transplantation. Arthroscopy.

[15] Gregush RV, Ferkel RD (2010). Treatment of the unstable ankle with an osteochondral lesion. Results and long-term follow-up. Am J Sports Med.

[16] Guhl JF, Parisien JS, Guhl JF, Boynton MD, Parisien JS (2004). Osteochondral pathology. Foot and ankle arthroscopy.

[17] Karlsson J, Peterson L (1991). Evaluation of ankle joint function: the use of a scoring scale. Foot.

[18] Kitaoka HB, Alexander IJ, Adelaar RS (1994). Clinical rating systems for the ankle-hindfoot, midfoot, hallux, and lesser toes. Foot Ankle Int.

[19] Kono M, Takao M, Naito K, Uchio Y, Ochi M (2006). Retrograde drilling for osteochondral lesions of the talar dome. Am J Sports Med.

[20] Lee KB, Bai LB, Chung JY, Seon JK (2010). Arthroscopic microfracture for osteochondral lesions of the talus. Knee Surg Sports Traumatol Arthrosc.

[21] Mei-Dan O, Carmont MR, Laver L, Mann G, Maffulli N, Nyska M (2012). Platelet-rich plasma or hyaluronate in the management of osteochondral lesions of the talus. Am J Sports Med.

[22] O’Loughlin PF, Heyworth BE, Kennedy JG (2010). Current concepts in the diagnosis and treatment of osteochondral lesions of the ankle. Am J Sports Med.

[23] Ogut T, Ayhan E, Irgit K, Sarikaya AI (2011). Endoscopic treatment of posterior ankle pain. Knee Surg Sports Traumatol Arthrosc.

[24] Saxena A, Eakin C (2007). Articular talar injuries in athletes: results of microfracture and autogenous bone graft. Am J Sports Med.

[25] Sefton GK (1979). Reconstruction of the anterior talofibular ligament for the treatment of the unstable ankle. J Bone Jt Surg [Br].

[26] Shearer C, Loomer R, Clement D (2002). Nonoperatively managed stage 5 osteochondral talar lesions. Foot Ankle Int.

[27] Steadman JR (1997). Microfracture technique for full-thickness chondral defects: technique and clinical results. Oper Tech Orthop.

[28] Taga I, Shino K, Inoue M, Nakata K, Maeda A (1993). Articular cartilage lesion in ankles with lateral ligament injury: an arthroscopic study. Am J Sports Med.

[29] Takao M, Uchio Y, Kakimaru H, Kumahashi N, Ochi M (2004). Arthroscopic drilling with debridement of remaining cartilage for osteochondral lesions of the talar dome in unstable ankles. Am J Sports Med.

[30] Takao M, Uchio Y, Naito K, Fukazawa I, Ochi M (2005). Arthroscopic assessment for intra-articular disorders in residual ankle disability after sprain. Am J Sports Med.

[31] Tegner Y, Lysholm J (1985). Rating systems in the evaluation of knee ligament injuries. Clin Orthop Rel Res.

[32] Thermann H, Becher C (2004). Microfracture technique for treatment of osteochondral and degenerative chondral lesions of the talus. 2-year results of a prospective study. Unfallchirurg.

[33] Valderrabano V, Leumann A, Frigg A, Pagenstert G, Wiewiorski M (2011). Autologous matrix-induced chondrogenesis-aided repair of osteochondral lesions of the talus. Tech Foot Ankle Surg.

[34] van Dijk CN, Mol BW, Lim LS, Marti RK, Bossuyt PM (1996). Diagnosis of ligament rupture of the ankle joint. Physical examination, arthrography, stress radiography and sonography compared in 160 patients after inversion trauma. Acta Orthop Scand.

[35] van Dijk CN, Reilingh ML, Zengerink M, van Bergen CJA (2010). Osteochondral defects in the ankle: why painful?. Knee Surg Sports Traumatol Arthrosc.

[36] Ventura A, Terzaghi C, Legnani C, Borgo E (2012). Arthroscopic four-step treatment for chronic ankle instability. Foot Ankle Int.

[37] Zengerink M, Struijs PA, Tol JL, van Dijk CN (2010). Treatment of osteochondral lesions of the talus: a systematic review. Knee Surg Sports Traumatol Arthrosc.

